# Risk of New Bloodstream Infections and Mortality Among People Who Inject Drugs With Infective Endocarditis

**DOI:** 10.1001/jamanetworkopen.2020.12974

**Published:** 2020-08-12

**Authors:** Charlie Tan, Esfandiar Shojaei, Joshua Wiener, Meera Shah, Sharon Koivu, Michael Silverman

**Affiliations:** 1 Department of Medicine, Schulich School of Medicine and Dentistry, London, Ontario, Canada; 2 Division of Infectious Diseases, St Joseph’s Health Care, London, Ontario, Canada; 3 Department of Epidemiology and Biostatistics, Schulich School of Medicine and Dentistry, London, Ontario, Canada; 4 currently a medical student at Schulich School of Medicine and Dentistry, London, Ontario, Canada; 5 Department of Family Medicine, Schulich School of Medicine and Dentistry, London, Ontario, Canada; 6 Division of Infectious Diseases, St Joseph’s Health Care and London Health Sciences Centre, London, Ontario, Canada

## Abstract

**Importance:**

People who inject drugs (PWID) who are being treated for infective endocarditis remain at risk of new bloodstream infections (BSIs) due to ongoing intravenous drug use (IVDU).

**Objectives:**

To characterize new BSIs in PWID receiving treatment for infective endocarditis, to determine the clinical factors associated with their development, and to determine whether new BSIs and treatment setting are associated with mortality.

**Design, Setting, and Participants:**

This retrospective cohort study was performed at 3 tertiary care hospitals in London, Ontario, Canada, from April 1, 2007, to March 31, 2018. Participants included a consecutive sample of all PWID 18 years or older admitted with infective endocarditis. Data were analyzed from April 1, 2007, to June 29, 2018.

**Main Outcomes and Measures:**

New BSIs and factors associated with their development, treatment setting of infective endocarditis episodes (ie, inpatient vs outpatient), and 90-day mortality.

**Results:**

The analysis identified 420 unique episodes of infective endocarditis in 309 PWID (mean [SD] patient age, 35.7 [9.7] years; 213 episodes [50.7%] involving male patients), with 82 (19.5%) complicated by new BSIs. There were 138 independent new BSIs, of which 68 (49.3%) were polymicrobial and 266 were unique isolates. Aerobic gram-negative bacilli (143 of 266 [53.8%]) and *Candida* species (75 of 266 [28.2%]) were the most common microorganisms. Ongoing inpatient IVDU was documented by a physician in 194 infective endocarditis episodes (46.2%), and 127 of these (65.5%) were confirmed by urine toxicology results. Multivariable time-dependent Cox regression demonstrated that previous infective endocarditis (hazard ratio [HR], 1.89; 95% CI, 1.20-2.98), inpatient treatment (HR, 4.49; 95% CI, 2.30-8.76), and physician-documented inpatient IVDU (HR, 5.07; 95% CI, 2.68-9.60) were associated with a significantly higher rate of new BSIs, whereas inpatient addiction treatment was associated with a significantly lower rate (HR, 0.53; 95% CI, 0.32-0.88). New BSIs were not significantly associated with 90-day mortality (HR, 1.76; 95% CI, 0.78-4.02); significant factors associated with mortality included inpatient infective endocarditis treatment (HR, 3.39; 95% CI, 1.53-7.53), intensive care unit admission (HR, 9.51; 95% CI, 4.91-18.42), and methicillin-resistant *Staphylococcus aureus* infective endocarditis (HR, 1.77; 95% CI, 1.03-3.03), whereas right-sided infective endocarditis was associated with a significantly lower mortality rate (HR, 0.41; 95% CI, 0.25-0.67).

**Conclusions and Relevance:**

In this study, new BSIs were common in PWID receiving parenteral treatment for infective endocarditis. Discharging patients to outpatient treatment was not associated with an increase in new BSI incidence or mortality; carefully selected PWID may therefore be considered for such treatment.

## Key Points

**Question:**

Among people who inject drugs and who are receiving treatment for infective endocarditis, what organisms are identified in new bloodstream infections, and is the inpatient vs outpatient treatment setting associated with increased risk of new bloodstream infections and mortality?

**Findings:**

In this cohort study that included 420 episodes of infective endocarditis in 309 people who inject drugs, new bloodstream infections complicated 82 episodes (20%) and were primarily caused by gram-negative bacilli and *Candida* species. New bloodstream infections and mortality were not more common in patients receiving outpatient treatment.

**Meaning:**

These findings suggest that new bloodstream infections are common in people who inject drugs being treated for infective endocarditis, but carefully selected, low-risk patients may be appropriate candidates for outpatient parenteral antimicrobial therapy.

## Teaser

This cohort study assesses new bloodstream infections in people who inject drugs (PWID) and are receiving treatment for infective endocarditis and investigates whether new infections and treatment settings are associated with mortality.

## Introduction

Infective endocarditis in people who inject drugs (PWID) is rising in incidence, coinciding with the opioid epidemic.^1,2,3,4,5,6^ Prolonged parenteral antimicrobial treatment is the standard of care for infective endocarditis in PWID. Although oral therapy has been shown to be effective for infective endocarditis in non-PWID,^7^ concerns regarding adherence have limited this approach in PWID. Outpatient parenteral antimicrobial therapy (OPAT) is safe, efficacious, and cost-effective for treating many infections,^8,9,10,11^ but PWID are generally not considered candidates. This decision is in part due to the risk of new bloodstream infections (BSIs) from ongoing intravenous drug use (IVDU), commonly through central venous catheters inserted for antimicrobial treatment.^9,12,13^ New BSIs are difficult to manage, with the selected antimicrobial regimen needing to treat both the initial infective endocarditis and the superimposed infection; they may also necessitate lengthening of antimicrobial therapy and hospital admission. Therefore, it is often mandated that infective endocarditis in PWID be treated in monitored inpatient settings to optimize adherence, deter IVDU, and prevent new BSIs. However, whether remaining admitted actually decreases IVDU or improves outcomes is unknown. Hence, recent Infectious Diseases Society of America guidelines for OPAT concluded that “there is insufficient evidence to make a recommendation for or against treating PWID with OPAT at home.”^14^^(p17)^

The objectives of this study were to characterize new BSIs in PWID receiving treatment for infective endocarditis and determine the clinical factors associated with their development. We also sought to compare the rates of new BSIs in inpatient and outpatient (ie, OPAT) settings and to determine whether new BSIs and treatment setting were associated with mortality.

## Methods

### Study Design

This retrospective cohort study was conducted across all 3 acute care hospitals in London, Ontario, Canada, from April 1, 2007, to March 31, 2018. Data were recently published on 202 first episodes of infective endocarditis in PWID presenting from April 1, 2007, to March 31, 2016.^15^ For the present analysis, we extended the time for enrollment and added recurrent episodes. Research ethics approval was obtained from the Lawson Research institutional review board. Informed consent from study participants was waived for this retrospective collection of deidentified data. This study adhered to the Strengthening the Reporting of Observational Studies in Epidemiology (STROBE) reporting guideline for cohort studies.^16^

### Patient Identification and Data Set Design

We included all PWID 18 years or older admitted during the study period who met modified Duke criteria for definite infective endocarditis.^17^ The hospital medical records were screened for patients who received a code from the *International Classification of Diseases, Ninth Revision, Clinical Modification* (*ICD-9-CM*), or *International Statistical Classification of Diseases, Tenth Revision, Clinical Modification* (*ICD-10-CM*), for infective endocarditis in their discharge diagnoses; the accuracy of these codes has been validated.^18^ Medical records were reviewed by 2 infectious diseases physicians (including E.S.), with data abstracted into a standardized data set. The data set was restricted to patients who met modified Duke criteria for definite infective endocarditis and reported active IVDU within 3 months of admission.^15^

The data set was organized into unique episodes of infective endocarditis, defined as occurring at least 6 months after a previous episode or associated with both a new vegetation and a new microorganism within 6 months.^15^ Each episode of infective endocarditis may consist of several admissions (eg, for complications, after leaving against medical advice), and individual patients may have multiple unique episodes. Follow-up was 90 days from the last hospital discharge associated with the episode, gathered from inpatient and outpatient health records (including physician visits, laboratory results, and radiography), pharmacy dispensing records, and local obituary records.

### Patient Characteristics

Demographic characteristics included age, sex, and homelessness; comorbidities included previous infective endocarditis, prosthetic cardiac valve, and HIV, hepatitis B virus, or hepatitis C virus infection. The microbiological origin of each infective endocarditis episode was determined by index blood cultures and cultures of operative specimens, when available. Endocardial involvement on echocardiography was documented. Variables for IVDU included substances used before admission, documented (by a physician in the medical record) and confirmed (by urine toxicology screen result) inpatient drug misuse, and referral to addictions treatment.

### Definition of BSIs

New BSIs were defined as the identification of a microorganism in blood culture not secondary to an infection at another body site (as per Centers for Disease Control and Prevention definitions for laboratory-confirmed BSIs^19^) and different from those grown on index blood cultures at the time of infective endocarditis diagnosis. The positive blood cultures must have been obtained at least 48 hours after index blood cultures and while the patient was receiving parenteral antimicrobials. They could not be associated with a new vegetation; these were classified as novel episodes of infective endocarditis. Single isolates of skin and oral microflora were excluded. We documented the microbiological origins and susceptibility profiles of new BSIs and whether they occurred during inpatient (≥48 hours after admission) or outpatient (≥48 hours after discharge) treatment.

### Outcomes and Hospital Care Measures

Outcomes included embolic complications (embolic stroke, intracerebral hemorrhage, mycotic aneurysm, pulmonary embolism, and intra-abdominal embolism), cardiac complications (heart failure, conduction delay, and intracardiac abscess), other infections (nonembolic central nervous system, respiratory, and musculoskeletal), acute kidney injury (≥1.5-fold elevation in creatinine level from baseline or need for renal replacement therapy), and hepatic injury (≥3-fold elevation in liver enzyme level from baseline). We also evaluated septic shock, intensive care unit (ICU) admission, in-hospital mortality, and 90-day mortality.

Hospital care measures included a peripherally inserted central catheter (PICC), PICC complications, and surgical management of infective endocarditis (ie, cardiac surgery). We determined whether patients received inpatient or outpatient treatment for infective endocarditis or left against medical advice. Patients who were treated predominantly as inpatients, defined as less than 14 days of outpatient antimicrobials or leaving against medical advice with less than 14 days of antimicrobials remaining, were considered to have received inpatient treatment.

We determined the total inpatient and outpatient days of intravenous access (central or peripheral) associated with each episode. This was determined via chest x-rays (for dates of central venous catheter insertion and removal), clinical notes, and medication administration records (with patients assumed to have intravenous access if administered an intravenous medication).

### Statistical Analysis

Data were analyzed from April 1, 2007, to June 29, 2018. We performed descriptive analyses of episodes of infective endocarditis with and without new BSIs, comparing demographic characteristics, comorbidities, microbiological origin, endocardial involvement, IVDU variables, complications, mortality, and hospital care measures. The analyses were repeated comparing episodes with and without new candidemia. Descriptive analyses were also performed comparing patients who died and who survived at 90 days after discharge. Variables for IVDU were not compared in mortality analyses because in-hospital IVDU and addiction treatment referral would not be possible for patients who died. Similarly, PICC use was not compared because PICCs were generally placed in clinically stable patients, with a citywide policy of requiring negative blood cultures before insertion. Discrete variables were coded dichotomously as present or absent (with additional categorizations where appropriate) and reported as frequencies and percentages. Continuous variables were reported as means with SDs. Missing data were documented for all variables. We also compared the incidence of new BSIs by treatment setting, defined as the number of BSIs per 1000 days of intravenous access, using Poisson regression.

We conducted multivariable time-dependent Cox proportional hazards regression analyses for clinical characteristics associated with new BSIs and 90-day mortality. The regression models included variables selected a priori based on expected clinical significance; both models were designed targeting at least 5 events for each variable.^20^ The model for new BSIs included previous infective endocarditis, inpatient treatment of infective endocarditis, physician-documented inpatient IVDU, PICC use, ICU admission, and inpatient addiction treatment. Time at risk was defined as time of admission until line removal at discharge or in the community. The model for 90-day mortality included new BSIs, inpatient treatment of infective endocarditis, right-sided infective endocarditis, methicillin-resistant *Staphylococcus aureus* (MRSA) infective endocarditis, ICU admission, surgical management, and inpatient addiction treatment referral. Time at risk was defined as from the time of admission until death or 90 days after discharge. To account for immortal time bias,^21^ new BSIs, inpatient addiction treatment, and surgical management were treated as time-dependent covariates. For both models, patients who left against medical advice were excluded owing to absence of appropriate follow-up; treatment setting was therefore a direct comparison between inpatient and outpatient treatment of infective endocarditis. All statistical analyses were performed with Excel, version 16.16.23 (Microsoft Corporation), and STATA statistical software, version 16.1 (StataCorp LLC). Two-sided *P* < .05 indicated significance.

## Results

### Description of Study Population

Our study included 420 unique episodes of infective endocarditis in 309 PWID (mean [SD] patient age, 35.7 [9.7] years; 213 episodes [50.7%] involving male patients and 207 [49.3%] involving female patients). We screened 2027 discharges with an *ICD-9-CM* or an *ICD-10-CM* code for infective endocarditis, identifying 688 episodes fulfilling definite modified Duke criteria. Of these, 268 were excluded for lack of IVDU within 3 months, yielding the final cohort. Twelve episodes were recurrences with a new organism within 3 months of a prior episode, although none occurred while patients still had an intravenous catheter. Two hundred fifty-three episodes (60.2%) involved inpatient treatment, 131 (31.2%) involved outpatient treatment, and 36 (8.6%) resulted in the patient leaving against medical advice.

The characteristics of the cohort are shown in Table 1. New BSIs were identified in 82 of 420 infective endocarditis episodes (19.5%; new BSI group). The predominant microbiological origin of all infective endocarditis episodes was *S aureus* (326 of 420 [77.6%]), with MRSA constituting 88 of 420 episodes (21.0%). New BSIs were more common among episodes in which the patient had a history of infective endocarditis (37 of 82 [45.1%] vs 91 of 338 [26.9%]). New BSIs were also more common among episodes with right-sided infective endocarditis (72 of 80 [90.0%] vs 229 of 329 [69.6%]); left-sided disease was more common among episodes without new BSIs (16 of 80 [20.0%] vs 125 of 329 [38.0%]).

**Table 1.** Demographic Characteristics, Site of Endocardial Involvement, and IVDU Variables of Infective Endocarditis Episodes, Stratified by New BSI

Variable	Episodes^a^		
	All (n = 420)	New BSI (n = 82)	No BSI (n = 338)
Demographic characteristics			
Age, mean (SD), y	35.7 (9.7)	34.5 (8.1)	36.0 (10.0)
Male	213 (50.7)	38 (46.3)	175 (51.8)
No fixed address	72 (17.1)	20 (24.4)	52 (15.4)
Comorbidities			
Previous infective endocarditis	128 (30.5)	37 (45.1)	91 (26.9)
Prosthetic valve	17 (4.0)	3 (3.7)	14 (4.1)
HIV	45 (10.7)	12 (14.6)	33 (9.8)
Receiving antiretroviral therapy^b^	23 (51.1)	7 (58.3)	16 (48.5)
Hepatitis B virus	2 (0.5)	0	2 (0.6)
Hepatitis C virus	318 (75.7)	66 (80.5)	252 (74.6)
Microbiological origin of infective endocarditis			
* Staphylococcus aureus*	326 (77.6)	67 (81.7)	259 (76.6)
Methicillin-sensitive *S aureus*	238 (56.7)	45 (54.9)	193 (57.1)
Methicillin-resistant *S aureus*	88 (21.0)	22 (26.8)	66 (19.5)
Viridans group streptococci	24 (5.7)	4 (4.9)	20 (5.9)
Nonviridans group streptococci	14 (3.3)	3 (3.7)	11 (3.3)
*Enterococcus* species	21 (5.0)	2 (2.4)	19 (5.6)
Coagulase-negative staphylococci	2 (0.5)	0	2 (0.6)
Nutritionally variant streptococci	1 (0.2)	0	1 (0.3)
HACEK	1 (0.2)	0	1 (0.3)
Other gram-negative bacilli	12 (2.9)	2 (2.4)	10 (3.0)
*Candida* species	11 (2.6)	2 (2.4)	9 (2.7)
Culture negative	8 (1.9)	2 (2.4)	6 (1.8)
Endocardial involvement			
Site of infection^c^			
Left-sided	141 (34.5)	16 (20.0)	125 (38.0)
Right-sided	301 (73.6)	72 (90.0)	229 (69.6)
Bilateral	33 (8.1)	8 (10.0)	25 (7.6)
Specific structure^c^			
Aortic valve	70 (17.1)	7 (8.8)	63 (19.1)
Mitral valve	83 (20.3)	10 (12.5)	73 (22.2)
Tricuspid valve	296 (72.4)	68 (85.0)	228 (69.3)
Pulmonic valve	6 (1.5)	2 (2.5)	4 (1.2)
Other native structure	14 (3.4)	7 (8.8)	7 (2.1)
Device-associated infection	2 (0.5)	0	2 (0.6)
No vegetation	11 (2.7)	2 (2.4)	9 (2.7)
IVDU variables			
Substance(s) used			
Opiate	365 (86.9)	80 (97.6)	285 (84.3)
Stimulant	272 (64.8)	55 (67.1)	217 (64.2)
Antidepressant	46 (11.0)	6 (7.3)	40 (11.8)
Polysubstance	321 (76.4)	72 (87.8)	249 (73.7)
Physician-documented inpatient drug misuse	194 (46.2)	69 (84.1)	125 (37.0)
Confirmed by urine screen	127 (30.2)	45 (54.9)	82 (24.3)
Inpatient prescription for opiates	402 (95.7)	82 (100)	320 (94.7)
Consultation with inpatient addictions treatment	156 (37.1)	35 (42.7)	121 (35.8)
Referral to outpatient addictions treatment^d^	151 (38.9)	35 (45.5)	116 (37.3)

Abbreviations: BSI, bloodstream infection; HACEK, *Haemophilus* species, *Aggregatibacter* species, *Cardiobacterium hominis*, *Eikenella corrodens*, and *Kingella* species; IVDU, intravenous drug use.

^a^ Unless otherwise indicated, data are expressed as number (percentage) of episodes.

^b^ Denominators include 45 episodes for all, 12 episodes in the new BSI group, and 33 episodes in the non-BSI group, accounting for patients who were HIV negative.

^c^ Denominators include 409 episodes for all, 80 episodes in the new BSI group, and 329 episodes in the non-BSI group, accounting for episodes without evidence of endocardial involvement on echocardiography but still fulfilling definite modified Duke criteria for infective endocarditis.

^d^ Denominators include 388 epidodes for all, 77 episodes in the new BSI group, and 311 episodes in non-BSI group, accounting for patients who died before discharge.

Physician-documented inpatient IVDU occurred in 194 of 420 infective endocarditis episodes (46.2%), whereas 127 of these (65.5%; 30.2% of all 420 episodes) had confirmed drug use by urine toxicology results. Drug use was associated with treatment setting; IVDU during admission was more frequent in episodes when patients remained in the hospital for the duration of antimicrobial therapy (120 of 253 [47.4%]) than in episodes when patients were discharged with OPAT (45 of 131 [34.4%]), with the highest rate in those who left against medical advice (29 of 36 episodes [80.6%]). Episodes complicated by new BSIs, compared with episodes without BSIs, had higher rates of physician-documented (69 of 82 [84.1%] vs 125 of 338 [37.0%]) and confirmed (45 of 82 [54.9%] vs 82 of 338 [24.3%]) drug use. Opiate (80 of 82 [97.6%] vs 285 of 338 [84.3%]) and polysubstance (72 of 82 [87.8%] vs 249 of 338 [73.7%]) use were also more common in the new BSI group (Table 1).

### Microbiological Origin of New BSIs

Among the 82 episodes of infective endocarditis complicated by new BSIs, 138 independent new BSIs with 266 unique isolates were noted (Table 2). Almost half of the new BSIs were polymicrobial (68 of 138 [49.3%]). Aerobic gram-negative bacilli were most frequent (143 of 266 [53.8%]), with high rates of resistance to penicillins and cephalosporins and many species with inducible AmpC β-lactamase activity. *Candida* species constituted 75 of 266 isolates (28.2%), with low rates of azole and amphotericin B resistance. Gram-positive bacteria constituted 48 of 266 isolates (18.0%), more than half of which were *Enterococcus* species. The susceptibility profiles of new BSIs caused by gram-negative bacteria, gram-positive bacteria, and *Candida* species are shown in eTables 1 to 3 in the Supplement. The antimicrobials used to treat new BSIs are shown in eTable 4 in the Supplement.

**Table 2.** Microbiological Origin of New BSIs, Stratified by Treatment Location

Organism	Isolates, No. (%)		
	All (N = 266)	Inpatient treatment (n = 196)	Outpatient treatment (n = 70)
Gram-positive bacteria	48 (18.0)	29 (14.8)	19 (27.1)
*Enterococcus* species	26 (9.8)	15 (7.7)	11 (15.7)
Vancomycin-resistant enterococcus	9 (3.4)	6 (3.1)	3 (4.3)
Viridans group and other α-hemolytic streptococci	13 (4.9)	7 (3.6)	6 (8.6)
Other	9 (3.4)	7 (3.6)	2 (2.9)
Gram-negative bacteria	143 (53.8)	107 (54.6)	36 (51.4)
*Pseudomonas* species	25 (9.4)	22 (11.2)	3 (4.3)
* Stenotrophomonas maltophilia*	22 (8.3)	21 (10.7)	1 (1.4)
*Acinetobacter* species	21 (7.9)	13 (6.6)	8 (11.4)
*Enterobacter* species	20 (7.5)	9 (4.6)	11 (15.7)
*Klebsiella* species	16 (6.0)	12 (6.1)	4 (5.7)
*Chryseobacterium* species	7 (2.6)	3 (1.5)	4 (5.7)
*Sphingomonas* species	6 (2.3)	4 (2.0)	2 (2.9)
*Serratia* species	5 (1.9)	5 (2.6)	0
Other	21 (7.9)	18 (9.2)	3 (4.3)
*Candida* species	75 (28.2)	60 (30.6)	15 (21.4)
* Candida albicans*	30 (11.3)	25 (12.8)	5 (7.1)
* Candida tropicalis*	19 (7.1)	14 (7.1)	5 (7.1)
* Candida dubliniensis*	11 (4.1)	8 (4.1)	3 (4.3)
* Candida glabrata*	9 (3.4)	9 (4.6)	0
* Candida parapsilosis*	4 (1.5)	4 (2.0)	0
* Candida lusitaniae*	1 (0.4)	0	1 (1.4)
Other yeast (not speciated)	1 (0.4)	0	1 (1.4)

Abbreviation: BSI, bloodstream infection.

### Outcomes and Hospital Care Measures

Episodes of infective endocarditis complicated by new BSIs, compared with episodes without BSIs, had higher rates of septic pulmonary emboli (60 of 82 [73.2%] vs 187 of 338 [55.3%]), whereas embolic strokes (6 of 82 [7.3%] vs 57 of 338 [16.9%]) and intra-abdominal emboli (11 of 82 [13.4%] vs 74 of 338 [21.9%]) were more frequent in episodes without new BSIs (non-BSI group) (Table 3). No other differences in embolic complications, cardiac complications, nonembolic infections, and end-organ damage were observed. Admission to the ICU was less frequent in the new BSI group (21 of 82 [25.6%] vs 127 of 338 [37.6%]). Overall 90-day mortality was similar between the new BSI (11 of 82 [13.4%]) and non-BSI groups (53 of 338 [15.7%]).

**Table 3.** Hospital Care Variables, Complications, and Outcomes of Infective Endocarditis Episodes, Stratified by New BSI

Variable	Episodes, No. (%)		
	All (N = 420)	New BSI (n = 82)	Non-BSI (n = 338)
Complications and outcomes			
Major embolic complications	330 (78.6)	64 (78.0)	266 (78.7)
Embolic stroke	63 (15.0)	6 (7.3)	57 (16.9)
Intracerebral hemorrhage	28 (6.7)	2 (2.4)	26 (7.7)
Mycotic aneurysm	20 (4.8)	2 (2.4)	18 (5.3)
Septic pulmonary embolism	247 (58.8)	60 (73.2)	187 (55.3)
Intra-abdominal embolism	85 (20.2)	11 (13.4)	74 (21.9)
Cardiac complications	97 (23.1)	19 (23.2)	78 (23.1)
Heart failure	76 (18.1)	18 (22.0)	58 (17.2)
Conduction delay	12 (2.9)	1 (1.2)	11 (3.3)
Myocardial or aortic root abscess	26 (6.2)	3 (3.7)	23 (6.8)
Nonembolic central nervous system infection	40 (9.5)	6 (7.3)	34 (10.1)
Bone and joint infection	64 (15.2)	12 (14.6)	52 (15.4)
Nonembolic respiratory infection	36 (8.6)	7 (8.5)	29 (8.6)
Acute kidney injury	78 (18.6)	15 (18.3)	63 (18.6)
Hepatic injury	10 (2.4)	2 (2.4)	8 (2.4)
Septic shock	145 (34.5)	24 (29.3)	121 (35.8)
ICU admission	148 (35.2)	21 (25.6)	127 (37.6)
In-hospital mortality	53 (12.6)	10 (12.2)	43 (12.7)
90-d mortality	64 (15.2)	11 (13.4)	53 (15.7)
Hospital care measures			
PICC	367 (87.4)	80 (97.6)	287 (84.9)
Inpatient treatment	253 (60.2)	70 (85.4)	183 (54.1)
Outpatient treatment	131 (31.2)	12 (14.6)	119 (35.2)
Left hospital against medical advice	36 (8.6)	0	36 (10.7)
Cardiac surgery	64 (15.2)	8 (9.8)	56 (16.6)

Abbreviations: BSI, bloodstream infection; ICU, intensive care unit; PICC, peripherally inserted central catheter.

Use of PICCs was more frequent among episodes complicated by new BSIs compared with episodes without BSIs (80 of 82 [97.6%] vs 287 of 338 [84.9%]) (Table 3); PICC complications are shown in eTable 5 in the Supplement. More episodes in the new BSI group involved inpatient treatment (70 of 82 [85.4%] vs 183 of 338 [54.1%]), whereas more episodes in the non-BSI group involved outpatient treatment (12 of 82 [14.6%] vs 119 of 338 [35.2%]) or involved the patient leaving against medical advice (0 of 82 vs 36 of 338 [10.7%]). Fewer episodes involved surgical management in the new BSI group (8 of 82 [9.8%]) compared with the non-BSI group (56 of 338 [16.6%]).

### Secondary Analysis of Episodes Complicated by Candidemia

New candidemia was identified in 55 of 420 infective endocarditis episodes (13.1%) and was associated with previous infective endocarditis (25 of 55 [45.5%] vs 103 of 365 [28.2%]), HIV (10 of 55 [18.2%] vs 35 of 365 [9.6%]), and right-sided infective endocarditis (49 of 54 [90.7%] vs 252 of 355 [71.0%]) (eTable 6 in the Supplement). Physician-documented (46 of 55 [83.6%] vs 148 of 365 [40.5%]) and urine toxicology–confirmed (31 of 55 [56.4%] vs 95 of 365 [26.0%]) drug use were more common in the new candidemia group (eTable 6 in the Supplement). With regard to outcomes, septic pulmonary emboli were more common among episodes with new candidemia (41 of 55 [74.5%] vs 206 of 365 [56.4%]); there was no difference in 90-day mortality (9 of 55 [16.4%] vs 55 of 365 [15.1%]) (eTable 7 in the Supplement). More episodes complicated by new candidemia were treated in the inpatient setting (49 of 55 [89.1%] vs 204 of 365 [55.9%]) (eTable 7 in the Supplement).

### Rates of Bloodstream Infections

The inpatient new BSI rate was 9.60 (95% CI, 7.95-11.5) BSIs per 1000 days of intravenous access, with 115 BSIs and 11 985 days of intravenous access. In comparison, the outpatient new BSI rate was 5.23 (95% CI, 3.50-7.46) BSIs per 1000 days of intravenous access, with 27 BSIs and 5165 days of intravenous access. The incidence rate ratio comparing inpatient with outpatient rates of new BSIs was 1.84 (95% CI, 1.20-2.90; *P* = .003).

### Variables Associated With BSIs

The multivariable Cox regression for predictors of new BSIs is shown in Table 4. Clinical factors associated with a significantly higher rate of new BSIs were previous infective endocarditis (hazard ratio [HR], 1.89; 95% CI, 1.20-2.98), inpatient treatment of infective endocarditis (HR, 4.49; 95% CI, 2.30-8.76), and physician-documented inpatient IVDU (HR, 5.07; 95% CI, 2.68-9.60). Inpatient addiction treatment was associated with a significantly lower rate of new BSIs (HR, 0.53; 95% CI, 0.32-0.88).

**Table 4.** Multivariable Cox Regression for Factors Associated With New BSIs

Variable	HR (95% CI)	*P* value
Previous infectious endocarditis	1.89 (1.20-2.98)	.006
Inpatient treatment of infectious endocarditis^a^	4.49 (2.30-8.76)	<.001
Physician-documented IVDU	5.07 (2.68-9.60)	<.001
Inpatient addiction treatment	0.53 (0.32-0.88)	.01
ICU admission	0.60 (0.35-1.02)	.06
PICC insertion	0.60 (0.14-2.56)	.49

Abbreviations: BSI, bloodstream infection; HR, hazard ratio; ICU, intensive care unit; IVDU, intravenous drug use; PICC, peripherally inserted venous catheter.

^a^ Compared with outpatient treatment of infectious endocarditis.

### Variables Associated With 90-Day Mortality

The multivariable Cox regression for variables associated with 90-day mortality is shown in Table 5. New BSIs were not significantly associated with 90-day mortality (HR, 1.76; 95% CI, 0.78-4.02). Clinical factors associated with a significantly higher 90-day mortality rate were inpatient treatment of infective endocarditis (HR, 3.39; 95% CI, 1.53-7.53), ICU admission (HR, 9.51; 95% CI, 4.91-18.42), and MRSA infective endocarditis (HR, 1.77; 95% CI, 1.03-3.03). Right-sided infective endocarditis (HR, 0.41; 95% CI, 0.25-0.67) was associated with a significantly lower 90-day mortality rate. Clinical characteristics associated with 90-day mortality are shown in eTable 8 in the Supplement.

**Table 5.** Multivariable Cox Regression for Factors Associated With 90-Day Mortality

Variable	HR (95% CI)	*P* value
New BSI	1.76 (0.78-4.02)	.18
Inpatient treatment of infective endocarditis^a^	3.39 (1.53-7.53)	.003
Right-sided infective endocarditis^b^	0.41 (0.25-0.67)	<.001
MRSA infective endocarditis	1.77 (1.03-3.03)	.04
ICU admission	9.51 (4.91-18.42)	<.001
Cardiac surgery	0.66 (0.27-1.61)	.36
Inpatient addiction treatment	0.64 (0.32-1.29)	.22

Abbreviations: BSI, bloodstream infection; HR, hazard ratio; ICU, intensive care unit; MRSA, methicillin-resistant *Staphylococcus aureus*.

^a^ Compared with outpatient treatment of infective endocarditis.

^b^ Compared with left-sided or bilateral infective endocarditis.

## Discussion

To our knowledge, this study is the largest to date describing PWID with infective endocarditis by detailed medical record review. New BSIs were a frequent complication, affecting 19.5% of infective endocarditis episodes. Previous infective endocarditis was associated with new BSIs, suggesting that PWID with entrenched addictions are at higher risk. The association between inpatient drug misuse and new BSIs suggests that ongoing IVDU was likely responsible for most new BSIs, indicating that inpatient addiction treatment may serve as a protective factor. Furthermore, in-hospital treatment of infective endocarditis was not associated with fewer new BSIs, and there was no association between new BSIs and complications or mortality.

Gram-negative bacteria were the most common cause of new BSIs, whereas gram-positive bacteria were cultured less frequently and were predominantly *Enterococcus* species; this is likely a reflection of almost all patients receiving antistaphylococcal and/or antistreptococcal therapy for their infective endocarditis. The new BSIs typically involved organisms that were resistant to patients’ infective endocarditis treatment. Gram-negative bacteria primarily consisted of the gram-negative components of ESKAPE organisms (*Enterococcus faecium*, *S aureus*, *Klebsiella pneumoniae*, *Acinetobacter baumannii*, *Pseudomonas aeruginosa*, and *Enterobacter* species)^22^; glucose nonfermenters; and organisms with inducible AmpC β-lactamase genes associated with antibiotic resistance, including *Pseudomonas* species, *Stentrophomonas maltophilia, Acinetobacter* species, *Enterobacter* species, and *Klebsiella* species. Candidemia was also common, particularly among inpatients; candidemia is classically associated with health care settings, but IVDU has emerged as an independent risk factor.^23^

Empirical therapy in patients with suspected new BSI would ideally include an agent with activity against *Pseudomonas* species and *S maltophilia*, the 2 most common gram-negative isolates, such as levofloxacin or an antipseudomonal carbapenem with trimethoprim-sulfamethoxazole. Vancomycin may be added if not already part of the infective endocarditis regimen to cover *Enterococcus* species. Empirical antifungal therapy would include fluconazole or an echinocandin.

Inpatient treatment of infective endocarditis was associated with increased rates of new BSIs and 90-day mortality compared with outpatient treatment. These findings may in part reflect detection bias, with admitted patients followed up more closely with serial clinical and microbiological assessments. However, follow-up to 90 days was comprehensive, including medical records capturing admissions and outpatient visits to all London hospitals and neighboring hospitals in southwestern Ontario, inpatient and outpatient pharmacy medication dispensing records, laboratory investigations (including blood cultures), radiography at regional inpatient and outpatient facilities, and local obituary records. Patients receiving OPAT also receive nursing visits and are evaluated by an infectious disease specialist at the end of treatment. This thorough assessment did not reveal any increase in new BSIs or mortality that would preclude PWID from OPAT, providing support that OPAT may be appropriate for carefully selected PWID. The development of long-acting antibiotics with broad gram-positive coverage including against MRSA (eg, dalbavancin, ortivancin) may also allow for safer delivery of OPAT to PWID in the future.^24^

We found that ongoing inpatient IVDU was common, documented by a physician in almost half the episodes of infective endocarditis; this is likely an underestimate given that data collection was retrospective. We could not identify any previous studies evaluating physician-reported IVDU in hospitalized PWID, although a study of self-reported inpatient drug use found a similarly high rate.^25^ However, fewer than one-third of our patients were referred to inpatient addiction treatment. Poor access to addictions support for hospitalized PWID is a widespread concern.^26,27^ Similarly, provision of harm reduction materials for inpatients is uncommon^28^ and not practiced at our study hospitals. Although PWID with infective endocarditis tend to be younger and have fewer comorbidities than patients with non–IVDU-associated infective endocarditis, they often have worse outcomes, including higher rates of readmission and mortality.^29,30,31,32,33^ These disparities are largely due to inadequate treatment of substance use disorders and limited harm reduction services^29,30,31,32,33^; such interventions therefore need to be prioritized for hospitalized PWID.

Previous studies have differed in whether OPAT is safe and effective in PWID. However, the studies tended to have small sample sizes and did not evaluate new BSIs as a complication. A literature review of 10 studies on OPAT in PWID^34^ found high treatment completion, low mortality, and few catheter-related complications, comparable to rates in non-PWID populations. Another study^35^ implemented a 9-point tool to identify PWID candidates for OPAT, with low-risk PWID discharged to complete treatment as outpatients. Implementation resulted in reduced length of stay and cost savings without increased readmissions. Similarly, a pilot randomized trial that combined OPAT with buprenorphine opioid agonist therapy found a significantly reduced length of hospital stay by 23.5 days.^36^ In contrast, a 2017 study found that 61.2% (41 of 67) of PWID discharged to receive OPAT experienced treatment failure.^37^ However, failure was defined broadly, including readmission, prolongation of antibiotic regimens, nonadherence to antibiotic regimens and clinic follow-up, and death. Furthermore, there was no comparator group, and 68.7% (46 of 67) of patients were discharged to a nursing facility, potentially indicating a population with high levels of comorbidities.

Although evidence suggests that oral antibiotics can be used to treat infective endocarditis,^7^ this practice has not been evaluated in PWID. If validated, oral treatment may represent an alternative to OPAT, eliminating the need for prolonged venous access and consequent risk of new BSIs.

### Strengths and Limitations

Our study has several strengths. This study is the first, to our knowledge, of PWID receiving long-term intravenous antimicrobial therapy to describe BSIs and compare outcomes by treatment setting. Our outcome measures were robust, and all cases were reviewed by infectious disease physicians and fulfilled modified Duke criteria for definite infective endocarditis. Comprehensive region-wide records allowed detailed follow-up of all patients to 90 days after discharge. We also accounted for immortal time bias by incorporating time-dependent variables.

Our study also has some limitations. First, owing to retrospective data collection, the accuracy of data depended on the fidelity of prior documentation. For example, data on nonfatal drug overdoses were limited, available for only 4 outpatients and 2 inpatients. Second, outcomes may have been underestimated in patients who received OPAT, with admitted patients monitored more closely (ie, detection bias). Third, our comparison of infective endocarditis treatment setting was nonrandomized, with those receiving outpatient infective endocarditis treatment likely having lower-risk IVDU behaviors and fewer comorbidities (ie, selection bias). However, our intention was not to prove the superiority of outpatient treatment, but to demonstrate that discharging selected PWID with OPAT was not associated with increased morbidity and mortality. Last, our results may not be generalizable to different OPAT programs. In London, OPAT involves parenteral antimicrobials administered at the patient’s residence, whereas OPAT at some other centers involves patients presenting to an infusion clinic, with attendance potentially compromised in PWID.

## Conclusions

We described new BSIs in PWID receiving treatment for infective endocarditis, finding higher frequency in patients with entrenched addictions and in the inpatient setting. Aerobic gram-negative bacilli and *Candida* species were the most common causative microorganisms. Ongoing IVDU was documented in almost half of inpatients being treated for infective endocarditis. Although our study was affected by detection and selection biases, we did not find higher rates of new BSIs or mortality among PWID discharged with OPAT, suggesting that OPAT may be appropriate for selected low-risk PWID. Prospective randomized studies of OPAT vs inpatient treatment or oral treatment for IVDU-associated infections are required. Furthermore, improved access to inpatient addictions and harm reduction services should be prioritized.

## Notes

Supplement. **eTable 1.** Susceptibility Profiles of New Bloodstream Infections Caused by Gram-Negative Bacteria **eTable 2.** Susceptibility Profiles of New Bloodstream Infections Caused by Candida Species **eTable 3.** Susceptibility Profiles of New Bloodstream Infections Caused by Gram-Positive Bacteria **eTable 4.** Antimicrobials Used to Treat New Bloodstream Infections **eTable 5.** Peripherally Inserted Central Catheter–Related Complications, Stratified by Treatment Location **eTable 6.** Demographic Characteristics, Site of Endocardial Involvement, and Intravenous Drug Use Variables of Infective Endocarditis Episodes, Stratified by New Candidemia **eTable 7.** Hospital Care Variables, Complications, and Outcomes of Infective Endocarditis Episodes, Stratified by New Candidemia **eTable 8.** Clinical Characteristics, Microbiologic Etiology, Site of Endocardial Involvement, Complications, and Hospital Care Variables of Infective Endocarditis Episodes, Stratified by 90-Day Mortality
