# Supplementary material for: Risk of New Bloodstream Infections and Mortality Among People Who Inject Drugs With Infective Endocarditis
Source: JAMA Netw Open. 2020 Aug 12;3(8):e2012974. doi: 10.1001/jamanetworkopen.2020.12974 (PMC7424403; doi:10.1001/jamanetworkopen.2020.12974)
Supplement: Supplement. — eTable 1. Susceptibility Profiles of New Bloodstream Infections Caused by Gram-Negative Bacteria eTable 2. Susceptibility Profiles of New Bloodstream Infections Caused by Candida Species eTable 3. Susceptibility Profiles of New Bloodstream Infections Caused by Gram-Positive Bacteria eTable 4. Antimicrobials Used to Treat New Bloodstream Infections eTable 5. Peripherally Inserted Central Catheter–Related Complications, Stratified by Treatment Location eTable 6. Demographic Characteristics, Site of Endocardial Involvement, and Intravenous Drug Use Variables of Infective Endocarditis Episodes, Stratified by New Candidemia eTable 7. Hospital Care Variables, Complications, and Outcomes of Infective Endocarditis Episodes, Stratified by New Candidemia eTable 8. Clinical Characteristics, Microbiologic Etiology, Site of Endocardial Involvement, Complications, and Hospital Care Variables of Infective Endocarditis Episodes, Stratified by 90-Day Mortality [file jamanetwopen-3-e2012974-s001.pdf]

Supplementary Online Content

Tan C, Shojaei E, Wiener J, Shah M, Koivu S, Silverman M. Risk of new bloodstream infections and mortality among people who inject drugs with infective endocarditis. *JAMA Netw Open*. 2020;3(8):e2012974. doi:10.1001/jamanetworkopen.2020.12974

eTable 1. Susceptibility Profiles of New Bloodstream Infections Caused by Gram-Negative Bacteria
eTable 2. Susceptibility Profiles of New Bloodstream Infections Caused by Candida Species
eTable 3. Susceptibility Profiles of New Bloodstream Infections Caused by Gram-Positive Bacteria
eTable 4. Antimicrobials Used to Treat New Bloodstream Infections
eTable 5. Peripherally Inserted Central Catheter–Related Complications, Stratified by Treatment Location
eTable 6. Demographic Characteristics, Site of Endocardial Involvement, and Intravenous Drug Use Variables of Infective Endocarditis Episodes, Stratified by New Candidemia
eTable 7. Hospital Care Variables, Complications, and Outcomes of Infective Endocarditis Episodes, Stratified by New Candidemia
eTable 8. Clinical Characteristics, Microbiologic Etiology, Site of Endocardial Involvement, Complications, and Hospital Care Variables of Infective Endocarditis Episodes, Stratified by 90-Day Mortality

This supplementary material has been provided by the authors to give readers additional information about their work.

©2020 Tan C et al. *JAMA Network Open*.

eTable 1. Susceptibility Profiles of New Bloodstream Infections Caused by Gram-Negative Bacteria

**Organism**	**Number of isolates with sensitivity data**	Amoxicillin-clavulanate (%)	Cefazolin (%)	Ceftriaxone/ Cefotaxime (%)	Ceftazidime (%)	Piperacillin-tazobactam (%)	Fluoroquinolone (ciprofloxacin, levofloxacin) (%)	TMP-SMX (%)	Carbapenem (imipenem, meropenem) (%)	Aminoglycoside (gentamicin, tobramycin) (%)
Pseudomonas spp.	24				20/21 (95.2)	20 (83.3)	23 (95.8)		20 (83.3)	21 (87.5)
Stenotrophomonas maltophilia	22				3/21 (14.3)		20 (90.9)	22 (100)		
Acinetobacter spp.	18				15 (83.3)	12 (66.7)	17 (94.4)	16 (88.9)	17 (94.4)	17 (94.4)
Enterobacter spp.	18			16 (88.9)***		15 (83.3)	16 (88.9)	17 (94.4)	17 (94.4)	17 (94.4)
Klebsiella spp.	16	13 (81.3)	12 (75)	13 (81.3)		13 (81.3)	13 (81.3)	10 (62.5)	15 (93.8)	14 (87.5)
Sphingomonas spp.	6				4/5 (80.0)	5 (83.3)	4 (66.6)	3/4 (75.0)	6 (100)	4 (66.6)
Serratia spp.	5			5 (100)***		4/4 (100)	5 (100)	5 (100)	5 (100)	5 (100)
Chryseobacterium spp.	4			0 (0)		2 (50.0)	4 (100)		2 (50.0)	1 (25.0)
Achromobacter spp.	3			0 (0)	1/1 (100)	3 (100)	0 (0)		3 (100)	0 (0)
Citrobacter spp.	3			2 (66.6)***		3 (100)	2 (66.6)	0 (0)	3 (100)	2 (66.6)
Veillonella spp.*	3									
Pantoea spp.	2			1/1 (100)		2 (100)	2 (100)	2 (100)	2 (100)	2 (100)
Brevundimonas spp.	1				0 (0)	1 (100)	0 (0)		1 (100)	1 (100)
Eikenella spp.**	1									
Escherichia coli	1	0 (0)	1 (100)	1 (100)		1 (100)	1 (100)		1 (100)	1 (100)
Raoultella spp.	1	1 (100)	1 (100)	1 (100)		1 (100)	1 (100)	1 (100)	1 (100)	1 (100)
Unspecified non-fermenting organism	1				1 (100)	1 (100)	0 (0)		1 (100)	0 (0)
Other unspecified gram negative bacteria	2	0 (0)	1 (50.0)	1 (50.0)		2 (100)	2 (100)	0 (0)	2 (100)	1 (50.0)

*Routine susceptibility testing not performed for *Veillonella* spp. due to high beta-lactam resistance.
**Routine susceptibility testing not performed for *Eikenella* spp. due to routine susceptibility to penicillins.
***Not recommended for therapy due to inducible *AmpC* β-lactamase activity.

©2020 Tan C et al. *JAMA Network Open*.

eTable 2. Susceptibility Profiles of New Bloodstream Infections Caused by *Candida* Species

Organism	Number of isolates with sensitivity data	Fluconazole (%)	Amphotericin B (%)	Caspofungin (%)
Candida albicans	26	26 (100)	26 (100)	4/4 (100)
Candida tropicalis	19	19 (100)	19 (100)	3/3 (100)
Candida dubliniensis	10	10 (100)	9/9 (100)	1/1 (100)
Candida glabrata	9	6* (66.7)	9 (100)	1/2 (50.0)
Candida parapsilosis	3	3 (100)	3 (100)	
Candida lusitaniae	1	1 (100)	1 (100)	

*Susceptibility of *Candida glabrata* to fluconazole was dose-dependent.

©2020 Tan C et al. *JAMA Network Open*.

eTable 3. Susceptibility Profiles of New Bloodstream Infections Caused by Gram- Positive Bacteria

**Organism**	**Number of isolates with sensitivity data**	Penicillin (%)	Ampicillin (%)	Cloxacillin (%)	Cefazolin (%)	Ceftriaxone (%)	Vancomycin (%)	TMP-SMX (%)	Clindamycin (%)	Gentamicin synergy (%)	Ciprofloxacin (%)
Enterococcus spp.	26		6 (23.1)				17 (65.4)			12/14 (85.7)	
Viridans group and other alpha-hemolytic streptococci	9	1 (11.1)				2/5 (40.0)	7 (77.8)				
Bacillus cereus	1						1 (100)		1 (100)		1 (100)
Granulicatella spp.	1	0 (0)				0 (0)	1 (100)				
Gemella spp.*	1										
Methicillin-resistant *Staphylcoccus aureus*	1			0	0		1 (100)	1 (100)	1 (100)		
Coagulase-negative staphylococci	1			0	0		1 (100)	0	0		
Peptostreptococcus spp.*	1										
Group A streptococcus*	1										

*Routine susceptibility testing not performed due to susceptibility to penicillins.

©2020 Tan C et al. *JAMA Network Open*.

eTable 4. Antimicrobials Used to Treat New Bloodstream Infections

**Antimicrobial**	**Frequency (%)**
Fluconazole	38 (31.4)
Ciprofloxacin/Levofloxacin	19 (15.7)
Caspofungin	15 (12.4)
Vancomycin	12 (9.9)
Imipenem/Meropenem	7 (5.8)
Daptomycin	6 (5.0)
TMP-SMX	5 (4.1)
Ceftriaxone	4 (3.3)
Amphotericin B	3 (2.5)
Piperacillin-tazobactam	3 (2.5)
Meropenem	2 (1.7)
Linezolid	2 (1.7)
Ceftazidime	2 (1.7)
Amoxicillin/Ampicillin	2 (1.7)
Metronidazole	1 (0.8)
Cefuroxime	1 (0.8)
Colistin	1 (0.8)
**Total**	**121 (100)**

©2020 Tan C et al. *JAMA Network Open*.

eTable 5. Peripherally Inserted Central Catheter–Related Complications, Stratified by Treatment Location

Complication	Inpatient	Outpatient
Deep vein thrombosis	6	1
Exit site skin and soft tissue infection	3	0
Lumen occlusion	13	1
Superficial thrombophlebitis	1	0

©2020 Tan C et al. *JAMA Network Open*.

eTable 6. Demographic Characteristics, Site of Endocardial Involvement, and Intravenous Drug Use Variables of Infective Endocarditis Episodes, Stratified by New Candidemia

**Variable (%)**	**New candidemia (n=55)**	**No new candidemia (n=365)**
Demographic characteristics		
Mean age, in years (standard deviation)	34.2 (7.9)	35.9 (9.9)
Male	25 (45.5)	188 (51.5)
No fixed address	14 (25.5)	58 (15.9)
Comorbidities		
Previous infective endocarditis	25 (45.5)	103 (28.2)
Prosthetic valve	2 (3.6)	15 (4.1)
HIV	10 (18.2)	35 (9.6)
On antiretroviral therapy[a]	6 (60.0)	17 (48.6)
Hepatitis B	0 (0)	2 (0.5)
Hepatitis C	43 (78.2)	272 (74.5)
Microbiologic etiology of infective endocarditis		
Staphylococcus aureus	43 (78.2)	283 (77.5)
Methicillin-sensitive *S. aureus*	30 (54.5)	208 (57.0)
Methicillin-resistant *S. aureus*	13 (23.6)	75 (20.5)
Viridans group streptococci	4 (7.3)	20 (5.5)
Non-viridans group streptococci	3 (5.5)	11 (3.0)
Enterococcus spp.	1 (1.8)	20 (5.5)
Coagulase-negative staphylococci	0 (0)	2 (0.5)
Nutritionally variant streptococci	0 (0)	1 (0.3)
HACEK	0 (0)	1 (0.3)
Other gram negative bacilli	2 (3.6)	10 (2.7)
Candida spp.	1 (1.8)	10 (2.7)
Culture-negative	1 (1.8)	7 (1.9)
Endocardial involvement		
Site of infection[b]		
Left-sided	12 (22.2)	128 (36.1)
Right-sided	49 (90.7)	252 (71.0)
Bilateral	7 (12.7)	26 (7.3)
Specific structure[b]		
Aortic valve	5 (9.3)	64 (18.0)
Mitral valve	7 (12.7)	76 (21.4)
Tricuspid valve	47 (87.0)	249 (70.1)
Pulmonic valve	1 (1.9)	5 (1.4)
Other native structure	5 (9.3)	9 (2.5)
Device-associated infection	0 (0)	2 (0.6)
No vegetation	1 (1.8)	10 (2.7)
Intravenous drug use variables		
Substance(s) used		
Opiate	54 (98.2)	311 (85.2)
Stimulant	37 (67.3)	235 (64.4)
Antidepressant	5 (9.1)	41 (11.2)
Polysubstance	48 (87.3)	273 (74.8)
Physician-documented inpatient drug misuse	46 (83.6)	148 (40.5)
Confirmed by urine screen	31 (56.4)	96 (26.3)
Inpatient prescription for opiates	55 (100)	347 (95.1)

©2020 Tan C et al. *JAMA Network Open*.

Consultation to inpatient addictions treatment	22 (40.0)	134 (36.7)
Referral to outpatient addictions treatment[c]	23 (45.1)	128 (38.0)

[a] Denominators were 10 episodes (in new candidemia group) and 35 episodes (in no new candidemia group), accounting for patients who were HIV negative. [b] Denominators were 80 episodes (in new candidemia group) and 329 episodes (in no new candidemia group), accounting for episodes without evidence of endocardial involvement on echocardiography but still fulfilling definite modified Duke criteria for infective endocarditis. [c] Denominators were 51 episodes (in new candidemia group) and 337 episodes (in no new candidemia group), accounting for patients who died before discharge.

©2020 Tan C et al. *JAMA Network Open*.

eTable 7. Hospital Care Variables, Complications, and Outcomes of Infective Endocarditis Episodes, Stratified by New Candidemia

Variable (%)	New candidemia (n=55)	No new candidemia (n=365)
Complications and outcomes		
Major embolic complications	44 (80.0)	286 (78.4)
Embolic stroke	5 (9.1)	58 (15.9)
Intracerebral hemorrhage	1 (1.8)	27 (7.4)
Mycotic aneurysm	0 (0)	20 (5.5)
Septic pulmonary embolism	41 (74.5)	206 (56.4)
Intra-abdominal embolism	9 (16.4)	76 (20.8)
Cardiac complications	15 (27.3)	82 (22.5)
Heart failure	15 (27.3)	62 (17.0)
Conduction delay	0 (0)	12 (3.3)
Myocardial or aortic root abscess	2 (3.6)	24 (6.6)
Non-embolic central nervous system infection	4 (7.3)	36 (9.9)
Bone and joint infection	7 (12.7)	57 (15.6)
Non-embolic respiratory infection	7 (12.7)	29 (7.9)
Acute kidney injury	10 (18.2)	68 (18.6)
Hepatic injury	2 (3.6)	8 (2.2)
Septic shock	16 (29.1)	129 (35.3)
Intensive care unit admission	15 (27.3)	133 (36.4)
In-hospital mortality	9 (16.4)	44 (12.1)
90-day mortality	9 (16.4)	55 (15.1)
Hospital care measures		
PICC insertion	54 (98.2)	313 (85.8)
Inpatient treatment	49 (89.1)	204 (55.9)
Outpatient treatment	6 (10.9)	125 (34.3)
Left hospital against medical advice	0 (0)	36 (9.9)
Cardiac surgery	4 (7.3)	60 (16.4)

PICC = peripherally-inserted central catheter.

©2020 Tan C et al. *JAMA Network Open*.

eTable 8. Clinical characteristics, Microbiologic Etiology, Site of Endocardial Involvement, Complications, and Hospital Care Variables of Infective Endocarditis Episodes, Stratified by 90-Day Mortality

Variable (%)	Dead at 90 days (n=64)	Alive at 90 days (n=356)
Demographic characteristics		
Mean age, in years (standard deviation)	37.4 (9.6)	35.4 (9.7)
Male	38 (59.4)	175 (49.2)
No fixed address	9 (14.1)	63 (17.7)
Comorbidities		
Previous infective endocarditis	25 (39.1)	103 (28.9)
Prosthetic valve	9 (14.1)	8 (2.2)
HIV	7 (10.9)	38 (10.7)
Hepatitis B	0 (0)	2 (0.6)
Hepatitis C	42 (65.6)	273 (76.7)
Microbiologic etiology of infective endocarditis		
Staphylococcus aureus	44 (68.8)	282 (79.2)
Methicillin-sensitive *S. aureus*	26 (40.6)	212 (59.6)
Methicillin-resistant *S. aureus*	18 (28.1)	70 (19.7)
Viridans group streptococci	6 (9.4)	18 (5.1)
Non-viridans group streptococci	2 (3.1)	12 (3.4)
Enterococcus spp.	5 (7.8)	16 (4.5)
Coagulase-negative staphylococci	0 (0)	2 (0.6)
Nutritionally variant streptococci	0 (0)	1 (0.3)
HACEK	0 (0)	1 (0.3)
Other gram negative bacilli	1 (1.6)	11 (3.1)
Candida spp.	3 (4.7)	8 (2.2)
Culture-negative	1 (1.6)	7 (2.0)
Endocardial involvement		
Site of infection[a]		
Left-sided	35 (56.5)	105 (30.3)
Right-sided	36 (58.1)	265 (76.4)
Bilateral	9 (14.5)	24 (6.9)
Specific structure[a]		
Aortic valve	22 (35.5)	47 (13.5)
Mitral valve	18 (29.0)	65 (18.7)
Tricuspid valve	36 (58.1)	260 (74.9)
Pulmonic valve	1 (1.6)	5 (1.4)
Other native structure	0 (0)	14 (4.0)
Device-associated infection	0 (0)	2 (0.6)
No vegetation	2 (3.1)	9 (2.5)
Complications		
Major embolic complications	59 (92.2)	271 (76.1)
Embolic stroke	25 (39.1)	38 (10.7)
Intracerebral hemorrhage	10 (15.6)	18 (5.1)
Mycotic aneurysm	6 (9.4)	14 (3.9)
Septic pulmonary embolism	34 (53.1)	213 (59.8)
Intra-abdominal embolism	28 (43.8)	57 (16.0)
Cardiac complications	25 (39.1)	72 (20.2)
Congestive heart failure	22 (34.4)	55 (15.4)
Conduction delay	2 (3.1)	10 (2.8)
Myocardial or aortic root abscess	7 (10.9)	19 (5.3)

©2020 Tan C et al. *JAMA Network Open*.

Non-embolic central nervous system infection	6 (9.4)	34 (9.6)
Bone and joint infection	10 (15.6)	54 (15.2)
Non-embolic respiratory infection	10 (15.6)	26 (7.3)
Acute kidney injury	24 (37.5)	54 (15.2)
Hepatic injury	6 (9.4)	4 (1.1)
Septic shock	59 (92.2)	86 (24.2)
Intensive care unit admission	53 (82.8)	95 (26.7)
Hospital care		
Bloodstream infection	11 (17.2)	71 (19.9)
Inpatient	57 (89.1)	196 (55.1)
Outpatient	7 (10.9)	124 (34.8)
Left hospital against medical advice	0 (0)	36 (10.1)
Cardiac surgery	5 (7.8)	59 (16.6)

[a] Denominators were 62 episodes (in dead at 90 days group) and 347 episodes (in alive at 90 days group), accounting for episodes without evidence of endocardial involvement on echocardiography but still fulfilling definite modified Duke criteria for infective endocarditis.

©2020 Tan C et al. *JAMA Network Open*.
